# Psychological Distress, Fatigue and Quality of Life in Patients with Gastrointestinal Stromal Tumors

**DOI:** 10.11621/pir.2022.0201

**Published:** 2022-06-15

**Authors:** Edelmira Berenice Carbajal-López, Dehisy Marisol Juárez-García, Teresa de Jesús Sánchez-Jauregui, Absalón Espinoza-Velazco, German Calderillo-Ruiz, Rodrigo Salas-Benavides

**Affiliations:** a Autonomous University of Nuevo Leon, Mexico; b Mexican Social Security Institute, Mexico; c National Institute of Cancerology, Mexico; d Foundation GIST, Mexico

**Keywords:** Gastrointestinal stromal tumors (GIST), distress, fatigue, quality of life, cancer

## Abstract

**Background:**

Gastrointestinal stromal tumors (GIST) represent 1% of all gastrointestinal tumors and are included in the list of rare diseases.

**Objective:**

1) To evaluate levels of psychological distress, fatigue, and quality of life. 2) To identify the variables that most influence distress among Mexican patients with GIST.

**Design:**

A cross-sectional study was conducted with a consecutive sample of 100 patients with GIST, who completed the following questionnaires online: Hospital Anxiety and Depression Scale (HADS) as a measure of distress, Multidimensional Fatigue Inventory (MFI), and Quality of Life Questionnaire (QLQ C30).

**Results:**

Distress was present in 31% of patients. No association was found between distress and sociodemographic/clinical variables. The patients with distress demonstrated higher scores in all fatigue dimensions and, regarding quality of life, had more symptoms and were lower functioning. Distress was positively associated with all fatigue dimensions and with QLQ C30 symptoms. Negative associations were found between distress and QLQ C30 functioning dimensions. The predictors of psychological distress were general fatigue, reduced motivation, and emotional functioning.

**Conclusion:**

The percentage of patients with distress was akin to the levels found in patients with the most common types of cancer. Fatigue in patients with GIST should be evaluated and managed to improve distress levels.

## Introduction

Gastrointestinal stromal tumors (GIST) are mesenchymal tumors that originate from Cajal cells in the digestive tract. They are primarily located in the stomach, small intestine, esophagus, colon, and rectum (Calderillo-Ruiz et al., 2019). GIST represents 1% of all gastrointestinal tumors (National Cancer Institute, 2019) and is included in the list of rare diseases, defined in the United States as those conditions that affect less than 200,000 people (Genetic and Rare Diseases Information Center, 2020). Like with other types of cancer that are considered rare diseases, identifying causes and prevention strategies for GIST is a challenge for patients, caregivers, and doctors (American Cancer Society, 2020).

Due to their characteristics, rare cancers often involve numerous doctor visits and misdiagnoses. Consequently, a delay in diagnosis is more likely to occur, which may lead to more limited and less effective treatment options than for most common cancers. Furthermore, following diagnosis, patients and family members have more difficulty finding information about their type of cancer (American Cancer Society, 2017; Horick et al., 2017). These circumstances make patients vulnerable to mental health issues.

Distress is defined as a “multifactorial, unpleasant experience of a psychological (i.e., cognitive, behavioral, emotional), social, spiritual, and/or physical nature that may interfere with the ability to cope effectively with cancer, its physical symptoms, and its treatment” (Riba et al., 2019). It is necessary to identify the level and nature of distress in cancer patients in all stages of the disease, as this will allow for early intervention where required (Riba et al., 2019). The diagnosis of gastric cancer is associated with distress (Howell & Olsen, 2012), and patients with gastrointestinal tumors experiencing distress have a reduced survival rate (Whitaker et al., 2017).

The first-line treatment options for GIST are surgical resection and tyrosine kinase inhibitors (TKIs) (Nishida et al., 2016). Both treatment options have shown a significant increase in the overall survival of patients (Call et al., 2019). The side effects of these treatments include fatigue, which is one of the symptoms that generates more distress (Tantoy et al., 2017). Furthermore, the distress generated by cancer-related fatigue is higher than produced by fatigue in healthy individuals, and persistent fatigue affects the quality of life of patients (Berger et al., 2015).

Fatigue occurs in about 40% of patients treated with TKIs. Patients also report pain which interferes with their physical, psychological, and social activities and quality of life (Bang et al., 2019; Sodergren et al., 2014; Wiener et al., 2012). In a qualitative study by Fauske et al. (2020), it was found that GIST patients treated with TKIs presented symptoms of fatigue, a lack of energy in performing activities as a family, uncertainty due to drug resistance, and fear of premature death.

Fatigue is an incapacitating and relevant symptom that is not only associated with current TKI use, but also with psychological discomfort and compromised physical functioning. More severe fatigue has been associated with higher levels of distress and reduced physical functioning (Poort et al., 2016). These symptoms may be related to a higher risk of distress (Olson et al., 2019).

There are limited data on the state of physical and mental health of patients with rare cancers (Horick et al., 2017). So far, only a few studies have evaluated psychological aspects in patients with GIST (Custers et al., 2015; Wiener et al., 2012). Therefore, the objective of this study was to assess levels of distress, fatigue, and quality of life, and identify the variables that most influence distress among Mexican patients with GIST.

## Methods

### Participants

The study was cross-sectional and used a consecutive sample of patients with GIST recruited from 2016 to 2017. The inclusion criteria were having a confirmed diagnosis of GIST, being 18 years of age, having an email address, and to be receiving or having received treatment with TKIs. Exclusion criteria were having a psychiatric disorder, involvement in another psychological research at the time of recruitment, and previous history of cancer. The study was approved by the institutional committee of investigation and ethics [R-2016-1901-90].

### Procedure

Patients with GIST were identified by oncologists from different institutions and referred to the GIST Foundation where they were called and invited to be a part of the study. Patients who met the inclusion criteria were sent an email containing an informed consent form which, if responded to with agreement, was followed up with a link to the questionnaires on the SurveyMonkey platform. They did not receive any type of reward for their participation.

### Questionnaires

General data questionnaire. The questionnaire gathered sociodemographic details along with clinical and psychological data.

The Mexican adaptation of the Hospital Anxiety and Depression Scale (HADS) was used to evaluate distress. It consists of 12 items: 6 on the depression subscale and 6 on the anxiety subscale. Each item is rated on a 4-point scale from 0 to 3, with subscale scores ranging from 0 to 18. The total combined score of both subscales was used as a measure of distress. Cronbach’s alpha for the total scale was 0.86 (Galindo-Vázquez et al., 2015). In this study, the HADS obtained a Cronbach’s alpha of 0.83.

The Multidimensional Fatigue Inventory (MFI) is a 20-item self-report that measures fatigue in cancer patients. It is comprised of the following dimensions: general fatigue (α=0.84), physical fatigue (α=0.83), mental fatigue (α=0.77), reduced motivation (α=0.79), and reduced activity (α=0.83). Each scale contains four items rated on a 5-point scale and two indicative and two contraindicative elements for fatigue. The scores on each subscale range from 4 to 20 points, with a higher total score representing higher fatigue levels. The complete instrument had a Cronbach’s alpha of 0.84 (Smets et al., 1995). In this study, the following Cronbach’s alpha values ​were obtained for the MFI dimensions: general fatigue (α=0.79), physical fatigue (α=0.76), mental fatigue (α=0.81), reduced motivation (α=0.65) and reduced activity (α=0.77). For the complete scale Cronbach’s alpha was α= 0.91.

The Quality-of-Life Questionnaire (QLQ-C30) developed by the European Organization for Cancer Research and Treatment (EORTC) was used to assess quality of life. It includes five functional scales (physical, emotional, role, social, and cognitive functioning), a global scale of health/quality of life, three symptom scales (fatigue, pain, and nausea/vomiting), and individual items that assess additional symptoms. For all scales, scores range from 0 to 100. For the functional and global quality of life scales, higher scores mean a better level of functioning. For symptom-oriented scales, a higher score means more severe symptoms. Cronbach’s alpha values between 0.70 and 0.87 have been reported (Fayers et al., 2011). In this study, the values obtained for Cronbach’s alpha were 0.85 and 0.83, for functional scales and symptom scales, respectively.

### Statistical Analysis

Descriptive analyses of sociodemographic, clinical, and psychological variables were performed in the SPSS. A cutoff point of ≥10 for the total HADS score was used to identify the patients with psychological distress (Costa Requena et al., 2009). The X^2^ test was used to investigate the association between sociodemographic/clinical variables and distress, and the Student’s t-test was used to compare fatigue and quality of life between patients with and without distress. The variables that correlated with distress with r ≤ .500 (Pearson’s correlation) were treated as independent variables in a multiple regression analysis, which was carried out using the backward method to identify the most important variables to describe the variability of distress.

## Results

A total of 100 patients with GIST participated in the study. The ages of the participants ranged from 26 to 84 years; the average years of schooling was 12. *Table 1* shows the descriptive statistics for the clinical, sociodemographic, and psychological variables. Distress was found in 31 patients.

**Table 1:** Clinical, sociodemographic, and psychological characteristics (N=100)

	Frequency
GIST	
Stomach	76
Small intestine	23
Esophagus	1
Treatment	
Surveillance	50
Inhibitors	50
Stage	
Localized	69
Avanced	31
Sex	
Female	69
Male	31
Marital Status	
Single	12
Married	65
Divorced	8
Widower	15
Worker	
Yes	56
No	35
	**Mean (SD)**
Age	52.3(15.2)
Scholarship	12.1(4.0)
Monthly (mexican income peso ($))	8420.60 (7699.93)
HADS	
Distress	7.4(5.9)
MFI	
General Fatigue	11.1(4.5)
Physical Fatigue	11.8(4.6)
Reduced activity	10.0(4.5)
Reduced Motivation	7.4(3.5)
Mental Fatigue	8.3(4.3)
QLQ C30	
Physical functioning	85.1(15.9)
Rol functioning	87.1(20.7)
Emotional functioning	74.5(24.2)
Cognitive functioning	80.5(24.1)
Social functioning	76.3(28.2)
Fatigue	28.7(25.6)
Nausea, vomiting	11.3(18.4)
Pain	19.3(20.2)
Dyspnea	16.3(26.5)
Insomnia	32.6(36.3)
Appetite loss	10.6(21.1)
Constipation	15.3(22.4)
Diarrhea	15.0 (25.2)
Financial difficulties	33.0(34.2)
Global health/QLQ	76.7(20.1)

*Note. HADS= Hospital Anxiety and Depression Scale.*

*MFI= Multidimensional Fatigue Inventory.*

*QLQ= Quality of Life Questionnaire.*

The *X^2^* test revealed no association between sociodemographic/clinical variables and distress. *Table 2* shows the comparison analysis between patients who presented distress and those who did not. Significant differences were found in all the fatigue dimensions of the MFI, with higher scores among patients with distress. When comparing the functional scales of the QLQ C30 among patients with and without distress, significant differences were found in all dimensions, with lower scores obtained by patients with distress. On the symptom scales, patients with distress showed significantly higher scores (i.e., more severe symptoms).

**Table 2:** Comparison and Correlation Analysis

	Not Distressed n=69	Distressed n=31	T (98)	p value	Distress (p value) r
MFI	M(SD)				
General Fatigue	9.5(3.9)	14.5(3.6)	–6.1^a^	.000	–.615(.000)
Physical Fatigue	10.6(4.5)	14.5(3.5)	–4.1	.000	–.458(.000)
Reduced Activity	8.9(4.1)	12.5(4.4)	–3.8^a^	.000	–.388(.000)
Reduced Motivation	6.2(2.7)	10.1(3.8)	–5.7	.000	–.586(.000)
Mental Fatigue	6.8(3.2)	11.7(4.5)	–6.0	.000	–.608(.000)
QLQ C30					
Physical	87.7(15.6)	79.3(15.3)	2.5	.015	.348(.000)
Rol	91.5(15.2)	77.4(27.4)	3.2	.001	.223(.026)
Emotional	83.5(18.0)	54.3(24.2)	6.0^a^	.000	.695(.000)
Cognitive	85.5(20.1)	69.3(28.5)	3.2	.002	.325(.001)
Social	84.2(22.8)	58.6(31.2)	4.6	.000	.407(.000)
Global Health/QLQ	82.4(18.5)	63.9(17.7)	4.7^a^	.000	–.512(.000)
Fatigue	20.9(21.5)	46.2(25.8)	–4.7^a^	.000	.542(.000)
Nausea **v**omiting	8.2(14.1)	18.2(24.4)	–2.5	.000	.281(.005)
Pain	14.7(17.9)	29.5(21.3)	–3.3	.001	.422(.000)
Dyspnea	8.6(19.5)	33.3(32.2)	–4.7	.000	.388(.000)
Sleep	24.6(32.1)	50.5(39.9)	–3.4	.001	.335(.001)
Appetite **l**oss	6.7(16.7)	19.3(26.9)	–2.8	.005	.453(.000)
Constipation	10.1(19.2)	26.8(24.9)	–3.3^a^	.002	.367(.000)
Diarrhea	11.1(21.1)	23.6(31.2)	–2.3	.021	.212(.034)
Financial difficulties	27.0(32.4)	46.2(35.1)	–2.5^a^	.013	.093(.357)

*Note. MFI= Multidimensional Fatigue Inventory. QLQ= Quality of Life Questionnaire.*

**^a^** *the variances have not been assumed to be equal.*

As shown in Table 2, all variables except financial difficulties correlated significantly with distress. In the case of the MFI and symptoms dimensions of the QLQ, there was a positive correlation with distress. This indicates that the greater the fatigue and symptomatology, the greater the distress. The functioning and general quality of life dimensions of the QLQ were negatively correlated to distress.

In the regression analysis with distress as the dependent variable and general fatigue, reduced motivation, mental fatigue, emotional functioning, fatigue, and global scale of health/quality of life as independent variables, the following model was obtained (Table 3): general fatigue, reduced motivation, and emotional functioning explained 58% of the variance; the analysis of variance was significant (p < .001).

**Table 3:** Regression Analysis

Distress	B	SE	Beta
Model			
Constant	10.05	2.39	
General Fatigue	.331	.118	.252
Reduce Motivation	.301	.148	.181
Emotional Functioning	-.115	.020	-.469

Note. R= .766; R^2^ = .587; R^2^_aj_ = .574; F = 45.492; p .001; * p < .05; ** p < .01

## Discussion

The aim of this study was to evaluate the levels of distress, fatigue, and quality of life, and identify the predictors of distress in patients with GIST. Although several studies reported on the effects of anticancer treatments on quality of life, only four studies have evaluated psychological aspects in patients with GIST (Custers et al., 2015; Langenberg et al., 2019; Poort et al., 2016; Wiener et al., 2012).

The distress prevalence obtained in this study was lower than that previously reported in studies of GIST patients from the Netherlands, where 34% of patients had high levels of distress (Custers et al., 2015; Langenberg et al., 2019).

Regarding the MFI dimensions, scores on general fatigue, physical fatigue, and reduced activity all indicated the presence of fatigue and were similar to those reported for cancer patients and higher than those reported for the general population (Hinz et al., 2013; Singer et al., 2011).

As for the QLQ-C30 scores, the EORTC provides reference values for gastric cancer but not for GIST specifically. Compared with the results of gastric cancer patients, patients with GIST showed higher scores in all functional scales except for cognitive functioning, implying better functioning. In terms of the symptom scales, patients with GIST had lower scores, meaning less severe symptoms and a better quality of life, similar scores were obtained only for insomnia (Scott et al., 2008). When comparing our results with those of GIST patients in the Netherlands (Custers et al., 2015); Mexican patients had poorer functioning scores predominantly in emotional and social areas, and presented more issues with insomnia and financial difficulties.

It is important to note that the patients in our study were part of an institution where they were provided with support in the form of information regarding the disease and its treatment effects. This can contribute to improved quality of life and perhaps explain the observed scores in terms of functioning and symptoms. However, in the absence of specific psychological care, patients with GIST show lower scores in emotional and social functioning and similar levels of stress and fatigue relative to patients with other cancers.

Although this study did not find an association between sociodemographic variables and distress, other studies have demonstrated that there are greater levels of distress among younger, single, and female patients (Acquati et al., 2017; Hellstadius et al, 2017). Lower educational levels and more advanced stages of the disease have also been reported to be associated with higher levels of distress (Kim et al., 2017).

In this study, the greater the fatigue, the greater the distress. These results are similar to the those obtained in a study of gastrointestinal cancer patients, where higher severity of fatigue or lower energy levels were also associated with increased reported distress (Tantoy et al., 2018). Another study also found that fatigue, sleep disturbance, pain, and sadness were among the symptoms present in cancer patients who reported higher levels of distress (Mehnert et al., 2018).

This study also demonstrated that distress can be predicted by emotional functioning as assessed by QLQ C30, which includes aspects of worry, anxiety, irritability, and depression. Similarly, in a study with breast cancer patients, it was found that subjective stress mediated the relationship between emotional distress and fatigue (Levkovich et al, 2018).

The data obtained in this study provides relevant information about the predictors of distress in GIST patients and can help to design interventions that consider different dimensions, namely physical, cognitive, and emotional. Managing fatigue in its various dimensions is important as high fatigue has been reported to be a risk factor for distress in the 3 years following diagnosis (Alfonsson et al., 2016).

Interventions with cognitive-behavioral, mindfulness, and psychoeducational components have shown effects on emotional state, self-efficacy in managing fatigue, and quality of life in cancer survivors (Corbett et al., 2019).

## Conclusion

A total of 31% of Mexican patients with GIST experienced distress, which was mainly related to general fatigue levels, reduced motivation, and emotional functioning. Fatigue in patients with GIST should be evaluated and managed in order to improve their distress levels.

## Limitations

The results of this study should be analyzed according to the following limitations: First, the data was collected from a sample within a support foundation for GIST patients. Second, only patients with e-mail addresses were invited to participate, and the results cannot be generalized to other types of patients. Third, the data was collected through self-reporting, and the results may thus be affected by social desirability.
